# Phase I/II study of nivolumab with or without ipilimumab for treatment of recurrent small cell lung cancer (SCLC): CA209-032

**DOI:** 10.1186/2051-1426-3-S2-P376

**Published:** 2015-11-04

**Authors:** Matthew Taylor, Scott Antonia, Johanna Bendell, Emiliano Calvo, Dirk Jäger, Filippo de Braud, Patrick A Ott, M Catherine Pietanza, Leora Horn, Dung T Le, Michael A Morse, José A López-Martin, Paolo A Ascierto, Olaf Christensen, Jason S Simon, Chen-Sheng Lin, Joseph Paul Eder

**Affiliations:** 1 Oregon Health & Science University, Portland, OR, USA; 2 H. Lee Moffitt Cancer Center and Research Institute, Tampa, FL, USA; 3 Sarah Cannon Research Institute/Tennessee Oncology, PLLC, Nashville, TN, USA; 4 START Madrid, Centro Integral Oncológico Clara Campa, Madrid, Spain; 5 Heidelberg University Hospital, Heidelberg, Germany; 6 Istituto Nazionale dei Tumori, Milan, Italy; 7 Dana-Farber Cancer Institute, Boston, MA, USA; 8 Memorial Sloan Kettering Cancer Center, New York, NY, USA; 9 Vanderbilt-Ingram Cancer Center, Nashville, TN, USA; 10 The Sidney Kimmel Comprehensive Cancer Center at Johns Hopkins, Baltimore, MD, USA; 11 Duke University Medical Center, Durham, NC, USA; 12 Hospital Universitario 12 de Octubre, Madrid, Spain; 13 Istituto Nazionale Tumori Fondazione G. Pascale, Naples, Italy; 14 Bristol-Myers Squibb, Princeton, NJ, USA; 15 Yale Comprehensive Cancer Center, New Haven, CT, USA

## Background

Treatment options for SCLC after failing platinum-based (PLT) chemotherapy (CT) are limited. Combined blockade of programmed death-1 (PD-1) and cytotoxic T-lymphocyte antigen-4 (CTLA-4) immune checkpoint pathways has anti-tumor activity with a manageable safety profile. Nivolumab is a fully human IgG4 PD-1 immune checkpoint inhibitor approved for melanoma and squamous NSCLC in the US and for melanoma in the EU and Japan. Interim efficacy and safety of nivolumab +/- ipilimumab, a CTLA-4 checkpoint inhibitor, in pretreated SCLC patients are reported.

## Methods

Patients with progressive disease (PD) after PLT first-line treatment were eligible, regardless of platinum sensitivity, tumor PD-L1 expression, or number of prior CT regimens. Patients were randomized to nivolumab 3 mg/kg IV Q2W or nivolumab+ipilimumab (1+1 mg/kg or 1+3 mg/kg) IV Q3W for 4 cycles, followed by nivolumab 3 mg/kg Q2W. Primary objective was objective response rate (ORR). Additional objectives included safety, progression-free survival (PFS), overall survival (OS), and biomarker analysis.

## Results

Of 90 patients enrolled (nivolumab, n=40; nivolumab+ipilimumab, n=50 [nivolumab 1+ipilimumab 1, n=3; nivolumab 1+ipilimumab 3, n=47]), 53% had ≥2 prior regimens. Efficacy results for evaluable patients are shown (Table 1). 20% of patients in the nivolumab arm and 42% in the nivolumab+ipilimumab arms remain on treatment. Discontinuations due to treatment-related adverse events (TRAEs) occurred in 8% of nivolumab and 11% of nivolumab+ipilimumab patients. TRAEs (all grades) in ≥10% of patients included fatigue (18%), diarrhea (13%), nausea (10%), and decreased appetite (10%) with nivolumab; and diarrhea (23%), fatigue (21%), rash (21%), pruritus (19%), hypothyroidism (15%), hyperthyroidism (13%), nausea (13%), maculopapular rash (13%), and increased lipase (11%) with nivolumab 1+ipilimumab 3. Grade 3–4 TRAEs in ≥5% of patients occurred only in the nivolumab 1+ipilimumab 3 arm and included diarrhea (9%) and increased lipase (6%). Pneumonitis occurred in 2 patients in the nivolumab arm (grade 1–2) and 1 patient in the nivolumab 1+ipilimumab 3 arm (grade 3–4). One patient in the nivolumab 1+ipilimumab 3 arm had treatment-related myasthenia gravis with fatal outcome. Updated efficacy, safety, biomarker analysis, and case studies (responses in a patient with PLT-refractory disease and in a patient after crossover to nivolumab/ipilimumab) will be presented.

**Table 1:** 

	Nivolumab (n-40)	Nivolumab + Ipilimumab (n=46)^a^
ORR, %	18	17

Complete response, %	0	2.2

Partial response, %	18	15

Stable disease,^b^ %	20	37

Disease control rate, %	38	54

Progressive disease, %	53^c^	37

Death prior to first response assessment, %	10	6.5^d^

Not evaluable (no tumor assessment follow-up), %	0	2.2^e^

Median time to objective response (months)	1.6	2.2

Median DOR, months (95% CI) Range	NR 4.1-11+	6.9 (1.5 NR) 1,5-11,1+

DOR=duration of response; NR=not reached.

^a^4 of 50 patients did not reach first tumor assessment at database lock.

^b^Of 17 patients with stage disease in the nivolumab + ipilimumab arm, 7 had confirmed partial response after databse lock, resulting in updated ORR of 32.6%.

^c^1 patient had progressive disease in the spine, requiring surgery.

^d^1 patient died due to an unrelated adverse event, 1 patient died due to treatment-related myasthenia gravis, 1 patient died due to progressive disease.

^e^1 patient had an unrelated adverse event leading to permanent discontinuation, and had no post-baseline tumor assessment.

## Conclusions

In this PD-L1 unselected SCLC population with progression after PLT-CT, nivolumab monotherapy and nivolumab+ipilimumab were generally well tolerated with manageable toxicity. Rare severe toxicities will require close follow-up. Durable responses occurred with nivolumab monotherapy and in combination with ipilimumab.

## Trial Registry

Clinical Trial Number: NCT01928394.

